# Large Language Model Analysis of Reporting Quality of Randomized Clinical Trial Articles: A Systematic Review

**DOI:** 10.1001/jamanetworkopen.2025.29418

**Published:** 2025-08-28

**Authors:** Apoorva Srinivasan, Jacob Berkowitz, Nadine A. Friedrich, Sophia Kivelson, Nicholas P. Tatonetti

**Affiliations:** 1 Department of Computational Biomedicine, Cedars Sinai Medical Center, Los Angeles, California; 2 Cedars Sinai Cancer, Cedars Sinai Medical Center, Los Angeles, California

## Abstract

**Importance:**

Incomplete reporting in randomized clinical trials (RCTs) obscures bias and limits reproducibility. Manual audits for adherence to the Consolidated Standards of Reporting Trials (CONSORT) guideline cannot keep pace with publication volume.

**Objectives:**

To build and validate a zero-shot large-language-model (LLM) pipeline for automated CONSORT assessment and to map reporting quality over time, biomedical disciplines, and trial features.

**Design, Setting, and Participants:**

This systematic review included RCTs that were indexed on PubMed, available in English, open access, human-participant research, and published between MONTH 1966 to MONTH 2024. PubMed PDFs were converted to XML and linked with Semantic Scholar and ClinicalTrials.gov metadata. Chat GPT-4o-mini was tested on the 50-article CONSORT–Text Classification Model (CONSORT-TM) benchmark, checked by experts in 70 randomly sampled RCTs, and then applied to the full sample.

**Exposure:**

Publication year, biomedical discipline, funding source, trial phase, US Food and Drug Administration regulation, and oversight features.

**Main Outcomes and Measures:**

The LLM judged whether each of 21 CONSORT items was met. Primary outcomes were (1) model performance vs expert review (precision, recall, and macro F1 score) and (2) proportion of items reported.

**Results:**

Of 53 137 screened PDFs, 21 041 RCTs (median [IQR] publication year, 2014 [2003-2020]; 30 disciplines) were included, with a registry-linked subset of 1790 RCTs that had a median (IQR) planned enrollment of 210 (95-440) participants. In the 70-article validation set (2210 decisions) LLM outputs matched experts 91.7% of the time (2026 of 2210 decision); the macro F1 score on CONSORT-TM was 0.86 (95% CI, 0.84-0.87). Mean CONSORT compliance increased from 27.3% (95% CI, 27.0%-27.6%) in 1966 to 1990 to 57.0% (95% CI, 56.8%-57.2%) in 2010 to 2024. However, reporting critical elements remained uncommon, such as allocation-concealment mechanism (16.1% [95% CI, 15.6%-16.6%]) and external-validity discussion (1.6% [95% CI, 1.5%-1.8%]). Compliance varied across disciplines from 35.2% (95% CI, 34.8%-35.6%) in pharmacology to 63.4% (95% CI, 62.1%-64.7%) in urology and showed only negligible associations with clinical trial characteristics (all Cramer *V* <0.10).

**Conclusions and Relevance:**

In this systemic review of RCTs, a zero-shot LLM audited CONSORT adherence at scale, uncovering persistent reporting gaps and wide disciplinary variation across biomedical fields, underscoring the need for targeted editorial action to boost transparency and reproducibility.

## Key Points

**Question:**

Can a zero-shot large-language-model (LLM) framework accurately assess Consolidated Standards for Reporting Trials (CONSORT) reporting in randomized clinical trials (RCTs), and what trends does it uncover?

**Findings:**

In this systematic review, an LLM performed well on 50 benchmark RCTs and then assessed 21 041 open-access RCTs, published from 1966 to 2024. Overall CONSORT compliance rose from 27% before 1990 to 57% after 2010, yet key methodological items remained poorly reported; compliance varied widely by biomedical discipline (35%-63%).

**Meaning:**

These findings suggest that LLMs could enable scalable, reliable auditing of RCT reporting and highlight persistent gaps that journals and funders should target to improve research transparency and reproducibility.

## Teaser

This study uses a zero-shot large-language model to automatically assess compliance with the Consolidated Standards of Reporting Trials guideline for English-language randomized clinical trials published from 1966 to 2024.

## Introduction

Randomized clinical trials (RCTs) are the cornerstone of evidence-based medicine. They are used to make regulatory decisions and provide evidence for clinical guidelines and practice. Rigorous design and conduct, combined with complete and accurate reporting, are essential for fully leveraging the strengths of RCTs. However, many RCTs have methodological flaws, and results are often biased.^1^ Across RCTs, there is a major risk for inflated significance estimates and problems with randomization, allocation concealment, and blinding if certain procedures are not respected.^2,3^ Poor reporting is a persistent issue potentially obscuring biases, complicating replication efforts, and ultimately undermining the trustworthiness of biomedical science.^4,5^ Reporting information about trial methods (eg, sample size calculation, randomization, and masking) and results (eg, effect estimates) are necessary to assess validity for use in research, evidence synthesis, and clinical guideline generation. While complete reporting alone is not sufficient to ensure the research is rigorous and reproducible, it is necessary.

The Consolidated Standards of Reporting Trials (CONSORT) statement, first published in 1996 and updated in 2001 and 2010,^6,7,8,9^ comprises a 25-item checklist designed to improve completeness and consistency in trial reporting (eTable 1 in Supplement 1). While CONSORT has been widely endorsed by biomedical journals and extended to various trial designs and interventions,^10^ adherence remains inconsistent across the literature.

Traditional assessments of CONSORT compliance have relied on manual review of relatively small publication samples,^11^ limiting large-scale analysis. Automated approaches are needed for comprehensive evaluation, but previous attempts using rule-based algorithms or traditional machine learning performed poorly on this complex task.^12,13^ Large language models (LLMs) offer a promising alternative, with their ability to process complex language structures and capture reporting nuances.

**Figure 1.**
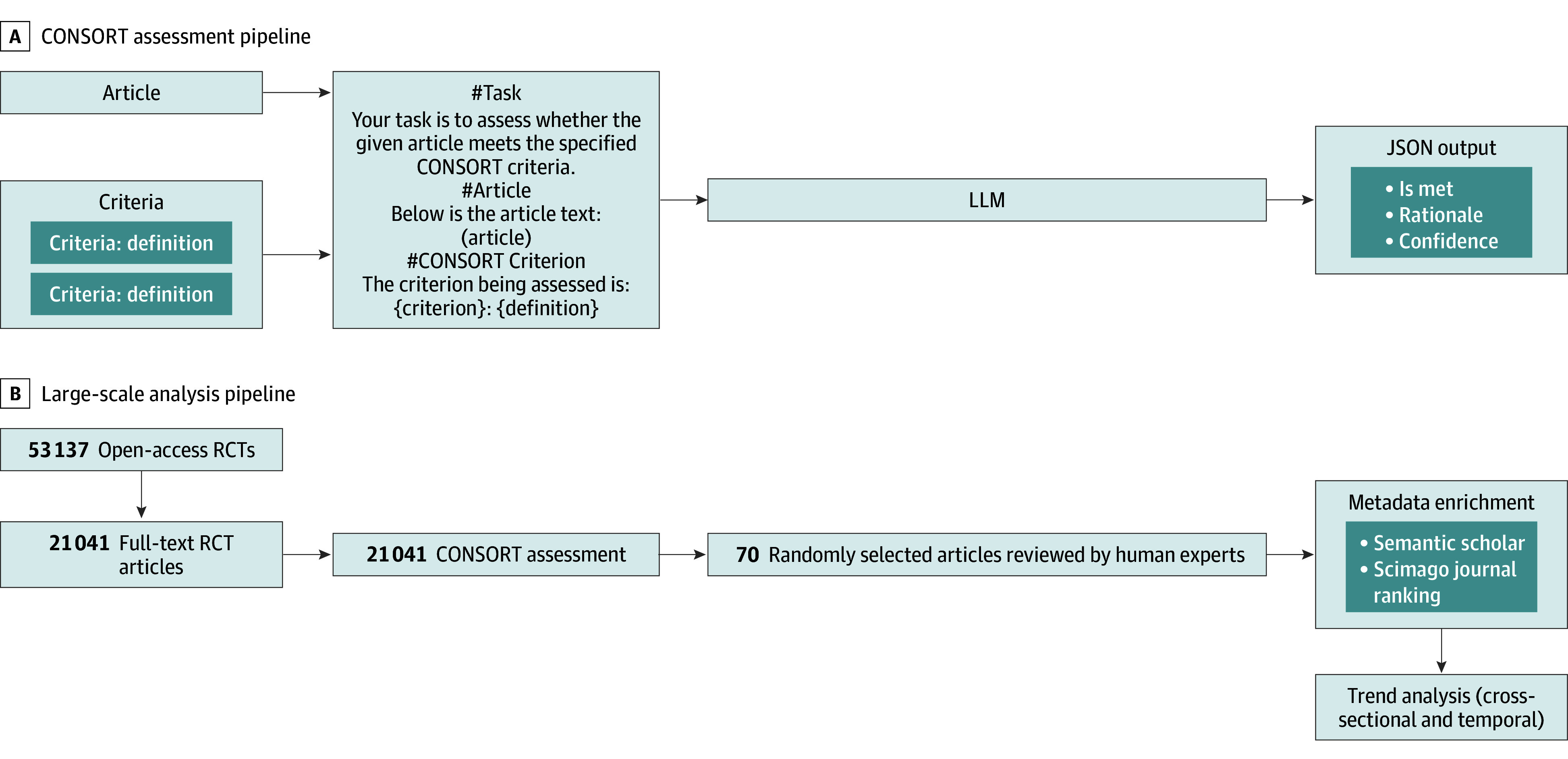
System Architecture for Automated Consolidated Standards of Reporting Trials (CONSORT) Compliance Assessment LLM indicates large language model; RCT, randomized clinical trial.

In this article, we leverage LLMs to evaluate CONSORT reporting in RCT publications at scale. We first validate our approach on the CONSORT–Text Classification Model (CONSORT-TM) dataset^14^ with expert review, then analyze reporting trends across a large number of RCTs. Our main contributions are developing a zero-shot LLM framework to achieve state-of-the-art performance on CONSORT compliance assessment, validated through expert review (Figure 1A), and conducting large-scale analysis of CONSORT reporting trends across a large sample of RCTs (Figure 1B), evaluating any changes in reporting over time and any persistent gaps in critical methodological details.

Prior natural language processing approaches to RCT analysis have primarily focused on PICO (Population, Intervention, Comparator, Outcome) classification,^15,16,17,18,19^ risk of bias assessment,^20,21^ and abstract classification.^22,23,24^ However, none have targeted comprehensive CONSORT guideline compliance.

Early CONSORT compliance evaluations used rule-based methods,^13^ while recent approaches employed bidirectional encoder representations from transformers–based models that showed improvements but required extensive labeled data.^25^ Initial LLM applications showed promise. One study reported encouraging results on 30 sports medicine articles,^26^ although a subsequent evaluation of the zero-shot capabilities of a later version of the same LLM on our target dataset achieved limited performance (F1 score, 0.51).^27^ Our work enhances these efforts through improved prompting techniques that leverage chain-of-thought reasoning.

Research on CONSORT adherence has traditionally relied on manual coding of small article samples across specific disciplines and time periods.^10,28,29,30,31,32,33,34,35^ While some larger studies have examined CONSORT references,^36,37^ they typically used simple keyword matching. Recent work has begun analyzing methods sections at scale,^25^ but comprehensive evaluation across all CONSORT items remains limited. To our knowledge, we present the first comprehensive assessment of full-text RCT articles using CONSORT guidelines on a dataset of this magnitude, enabling deeper insights into reporting quality trends across the clinical research landscape.

## Methods

### Dataset

For model evaluation, we used a previously curated dataset called the CONSORT-TM corpus^14^ for this study. It consists of 50 RCT publications annotated at the sentence level with 37 CONSORT checklist items.

For large-scale analysis, we identified 53 137 open-access human RCTs from PubMed (publication dates, 1966-2024) and obtained 21 041 full-text PDFs. Articles spanned 4 time periods: 1966 to 1990 (2771 articles), 1990 to 2000 (1969 articles), 2000 to 2010 (3765 articles), and 2010 to 2024 (10 447 articles). PDFs were converted to XML (PyMuPDF version 1.26.3) and enriched with metadata via Semantic Scholar. For a subset of 1790 articles, we extracted NCT numbers and obtained trial characteristics from ClinicalTrials.gov, including phase, funding source, US Food and Drug Administration (FDA) status, data monitoring committees, and safety outcomes. Only primary results articles were analyzed; protocols and rationale articles were not ingested (eMethods in Supplement 1).

### Models and Prompting

Three proprietary models (GPT-4 [LLM 1], GPT-4o [LLM 2], and GPT-4o-mini [LLM 3] [OpenAI]) were accessed through an Azure Health Insurance Portability and Accountability Act–compliant end point, and 1 open-source model (Llama-2-7B-chat [LLM 4]) was installed locally. All were used in zero-shot mode, as it offers the simplest path toward broader deployment.

Each criterion was assessed independently for every article. For each criterion, the entire article content was fed into the model, and the assessment was conducted one criterion at a time. (eFigure 1 in Supplement 1).

The output is a JSON string containing 4 elements: (1) criterion, ie, the specific criterion being assessed; (2) rationale, ie, step-by-step reasoning as to why the RCT does or does not meet the criterion; (3) decision, ie, output “MET” if the RCT meets the criterion or “NOT MET” if the RCT does not; and (4) confidence, ie, “Low,” “Medium,” or “High” confidence in decision. The rationale provided by the LLM was particularly important for model explainability, as it offered a detailed explanation of the decision-making process for each criterion. Self-reported confidence levels were used to assess the reliability of the model’s output.

### Model Evaluation

We evaluated the ability of our LLMs to determine whether an RCT article met or did not meet a set of inclusion criteria for each of the CONSORT items. The models were assessed based on their performance across standard binary classification metrics, including precision, recall, and both macro and micro F1 scores.

### Model Deployment

We applied our most efficient and best performing model to open access National Center for Biotechnology Information articles published between 1966 and 2024 to evaluate the quality of RCT reporting over time. The model was reprompted for each checklist item per article.

### Human Validation

To validate performance, we drew a stratified random sample of 70 articles from the 21 041-article corpus (11 published 1966-1990, 24 in 1990-2000, 13 in 2000-2010, and 22 in 2010-2024). Sampling ensured representation of each of the 30 Scimago specialties (≥2 articles each). For reporting, these specialties were collapsed into 5 broader groups: organ-system clinical care (36 articles), diagnostic and procedural support (17 articles), basic and translational science (8 articles), population and allied health (15 articles), and uncategorized (9 articles) (eTable 2 in Supplement 1).

Four reviewers (1 clinician [N.A.F], 3 data scientists [A.S., J.B., and S.K.]) independently judged every checklist decision provided by the LLMs as correct, partially correct, or incorrect. To quantify human reliability, a stratified subset of 10 articles was double-annotated; the Cohen κ of 0.64 indicated substantial agreement.

### Large-Scale Analysis

We examined (1) temporal trends across 4 publication eras, (2) discipline-level patterns (Scimago), and (3) trial-level factors from ClinicalTrials.gov. The trial-level factors included phase, funder, FDA status, region, safety monitoring, and adverse-event reporting.

### Statistical Analysis

Descriptive results are reported as counts and percentages or as medians with IQRs; 95% CIs for proportions were obtained with the Wilson method. Between-group differences in categorical variables (eg, medical discipline, FDA regulation) were examined with χ^2^ tests, and Fisher exact tests were substituted when any expected cell count was fewer than 5. Associations between continuous measures, such as reporting completeness percentage and citation counts, were assessed with Pearson correlation. For all categorical comparisons, the effect size was summarized with Cramer *V* and interpreted as negligible (<0.10), small (0.10-0.19), medium (0.20-0.29), or large (≥0.30). All subgroup analysis were decided a priori. All tests were 2-sided, and a *P* < .05 denoted statistical significance. Analyses were performed in Python version 3.8 (Python Software Foundation) with the pandas version 2.0, SciPy version 1.10, and statsmodels version 0.14 libraries.

## Results

A total of 21 041 open-access human RCT articles met the inclusion criteria and formed the analytic corpus. These included a total of 886 788 criteria items evaluated. The trials spanned 30 biomedical specialties and 4 publication eras (1966-1990, 1990-2000, 2000-2010, and 2010-2024). The median article was published in 2014 (IQR, 2003-2020). Among the 1790 articles with a ClinicalTrials.gov identifier, the median (IQR) planned enrolment was 210 (95-440) participants.

### Zero-Shot Criteria Assessment

All LLMs exceeded the previous state-of-the-art system by at least 40 percentage points in macro F1 score (Table).^27^ LLM 1 achieved the highest overall performance with a macro F1 score of 0.89 (95% CI, 0.88-0.90), precision of 0.93 (95% CI, 0.92-0.95), and accuracy of 0.84 (95% CI, 0.83-0.86). LLM 3 offered the best speed-to-accuracy trade-off with a macro F1 score of 0.86 (95% CI, 0.84-0.87) and precision of 0.97 (95% CI, 0.95-0.98) and was therefore selected for downstream analyses (item-wise performance appears in eTable 3 in Supplement 1). To ensure robustness, we repeated the zero-shot pipeline with LLM 3 three times and got identical results each time (eTable 4 in Supplement 1). LLM 4 model performed substantially less well (macro F1 score, 0.74 [95% CI, 0.72-0.76]), chiefly because of reduced recall.

**Table.** Zero-Shot Results on Consolidated Standards of Reporting Trials–Text Classification Model Dataset

Model	Estimate (95% CI)				
	Accuracy	Precision	Recall	Macro F1 score	Micro F1 score
LLM 1	0.84 (0.83-0.86)	0.93 (0.92-0.95)	0.85 (0.84-0.87)	0.89 (0.88-0.90)	0.84 (0.83-0.85)
LLM 2	0.78 (0.76-0.80)	0.94 (0.93-0.96)	0.75 (0.73-0.77)	0.84 (0.82-0.85)	0.78 (0.76-0.80)
LLM 3	0.81 (0.79-0.83)	0.97 (0.95-0.98)	0.77 (0.75-0.80)	0.86 (0.84-0.87)	0.81 (0.79-0.83)
LLM 4	0.67 (0.65-0.69)	0.90 (0.88-0.92)	0.63 (0.61-0.66)	0.74 (0.72-0.76)	0.67 (0.65-0.70)
Jiang et al,^27^ 2024	NA	0.48 (NA)	0.54 (NA)	0.51 (NA)	0.49 (NA)

Abbreviations: LLM, large language model; NA, not applicable.

### Confidence Filtering

LLM 3 self-reported confidence for every judgment (eTable 5 in Supplement 1). High-confidence decisions accounted for 804 593 of 886 788 items (90.8%) and showed excellent agreement with ground-truth labels (macro F1 score, 0.95 [95% CI, 0.93-0.96]; precision, 0.97 [95% CI, 0.96-0.98]; accuracy, 0.92 [95% CI, 0.90-0.93]). Medium-confidence decisions were markedly less reliable (macro F1, 0.31 [95% CI, 0.24-0.36]). No low-confidence outputs were produced. To maximize validity, all subsequent analyses used only high-confidence assessments; medium-confidence items were filtered out.

### Human Validation

Human expert evaluation of LLM 3 rationales for 70 articles showed 81.21% correct (1795 of 2210), 10.44% partially correct (231 of 2210), and 8.35% incorrect (184 of 2210) assessments. Error analysis revealed 2 main limitations: (1) misclassification of absent events (eg, no protocol changes) as unreported items and (2) incorrect inference of study locations from author affiliations (eFigure 2 in Supplement 1).

Based on human expert validation, we identified 4 CONSORT items that our model had difficulty accurately assessing: interim analyses and stopping guidelines (item 7b), changes to methods after trial commencement (item 3b), changes to trial outcomes (item 6b), and reasons for trial termination (item 14b). These items share a common characteristic—they report events that may not occur in every trial. Our model often misinterpreted the absence of these events as nonreporting rather than recognizing them as not applicable. Additionally, these items were infrequently reported across the dataset (reported in <5% of articles), limiting their impact on overall compliance scores. Therefore, we excluded these 4 items from our final analysis to ensure the reliability of our findings.

### Large-Scale RCT Reporting Quality Analysis

#### Overall CONSORT Item Reporting

**Figure 2.**
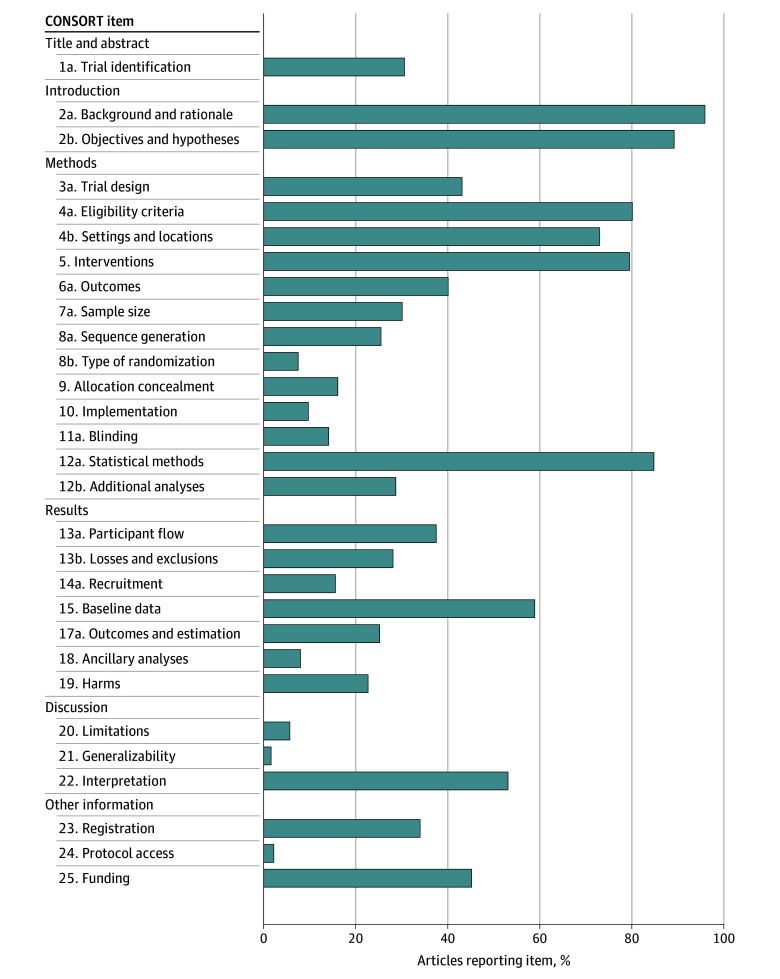
Consolidated Standards of Reporting Trials (CONSORT) Item Reporting Rates in 21 041 Randomized Clinical Trials

The most frequently reported CONSORT items were scientific background and rationale (20 175 of 21 041 trials [95.9%; 95% CI, 95.6%–96.1%]) and specific objectives and hypotheses (18 769 trials [89.2%; 95% CI, 88.8%-89.6%]). However, critical methodological details were often missing. Only 337 articles (1.6%; 95% CI, 1.5%-1.8%) discussed external validity; 3392 articles (16.1%; 95% CI, 15.6%-16.6%) described allocation mechanisms, and 468 articles (2.2%; 95% CI, 2.0%-2.4%) provided protocol access information (Figure 2).

#### Temporal Trends in Reporting

**Figure 3.**
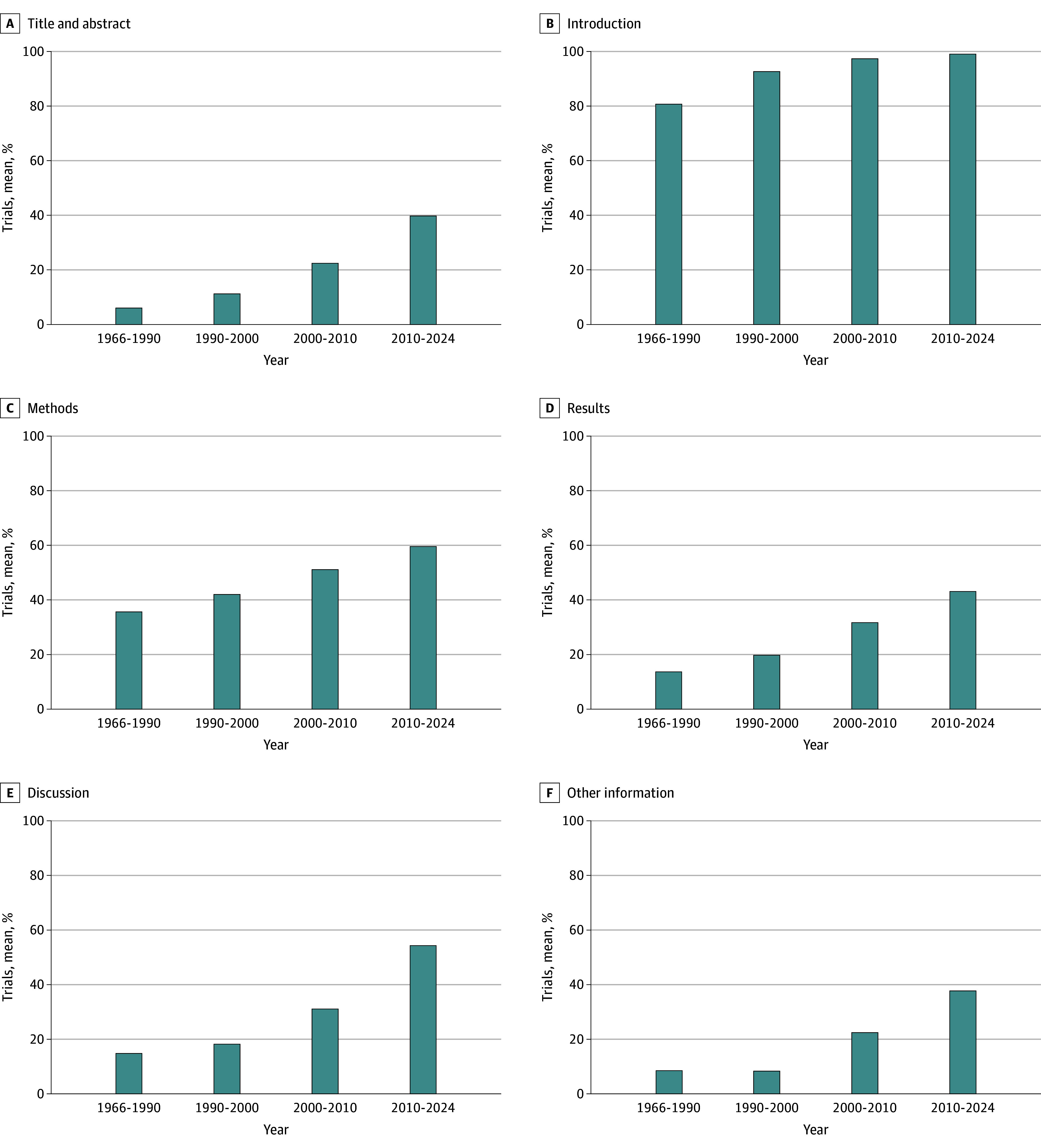
Reporting by Time Period and Article Section

We observed substantial improvement in CONSORT reporting rates over time (Figure 3). The mean compliance rate increased from 27.3% (19 727 of 72 279 items; 95% CI, 27.0%-27.6%) in 1966 to 1990 to 33.9% (18 556 of 54 704 items; 95% CI, 33.5%-34.3%) in 1990 to 2000, representing a 24.3% relative increase (*P* < .001). The most recent period (2010-2024) showed further improvement to 57.0% (195 385 of 342 873 items; 95% CI, 56.8%-57.2%), representing a 26.7% increase (*P* < .001). Despite these significant improvements across each time interval, the average proportion of CONSORT items reported remained below 60%, indicating persistent reporting gaps despite widespread guideline adoption.

### Variations by Field and Biomedical Discipline

**Figure 4.**
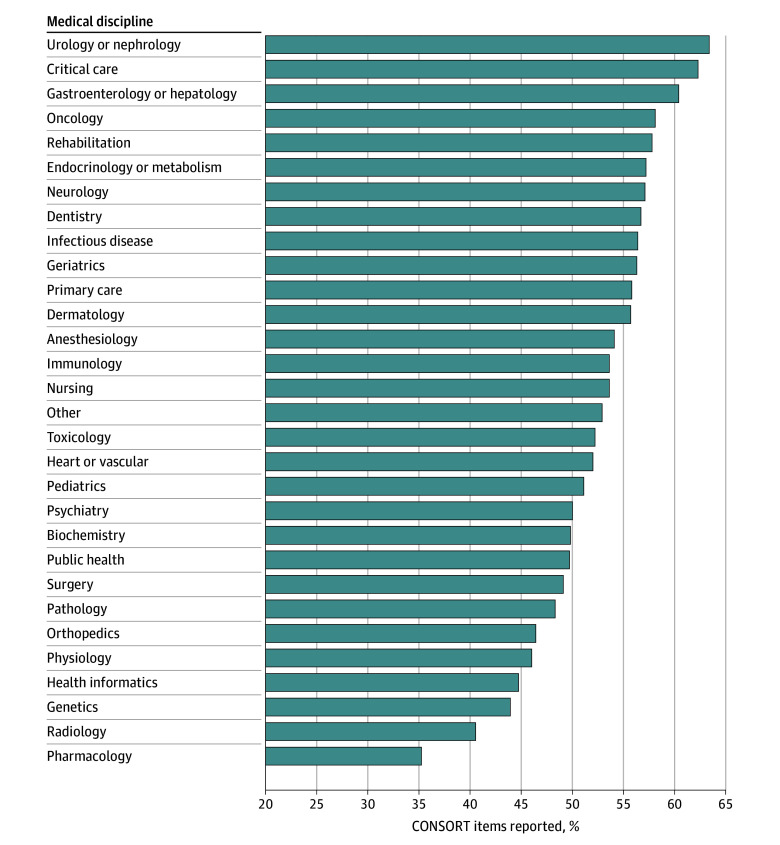
Consolidated Standards of Reporting Trials (CONSORT) Reporting by Medical Discipline

The reporting of CONSORT items varied significantly across medical disciplines (Figure 4). Urology and nephrology had the highest proportion of items met over the full-time interval measured (3146 of 4966 [63.4%; 95% CI, 62.1%-64.7%]), followed by critical care (1877 of 3014 [62.3%; 95% CI, 60.6%-64.0%]). On the lower end of the spectrum, pharmacology had the lowest reporting rates (27 616 of 78 542 items [35.2%; 95% CI, 34.8%-35.6%]), followed by radiology (668 of 1651 items [40.5%; 95% CI, 38.0%-42.8%]).

### Trial Characteristics and Oversight

#### Trial Phase and Funding

Early phase 1 (18 of 31 items [57.9%; 95% CI, 52.4%-63.2%]) and phase 1 (2562 of 4344 items [59.0%; 95% CI, 57.5%-60.4%]) trials had the lowest reporting rates, while phase 2 trials (5001 of 7513 items [66.6%; 95% CI, 65.5%-67.6%]) demonstrated the highest compliance (eFigure 3A in Supplement 1). By continent, European trials had the highest overall compliance (6868 of 10 220 items [67.2%; 95% CI, 66.0%-67.8%]), followed by North American trials (18 131 of 28 419 items [63.8%; 95% CI, 62.5%-64.4%]) (eFigure 3B in Supplement 1). Among major funders, federal agency–funded trials (366 of 562 items [65.1%; 95% CI, 61.1%-68.9%]) and industry-sponsored trials (10 020 of 15 747 items [63.6%; 95% CI, 62.9%-64.4%]) showed similar compliance rates, with no significant difference between them. (eFigure 3C in Supplement 1).

#### Regulatory Oversight and Safety Monitoring

Compliance was lower in FDA-regulated RCTs than in other trials (3827 of 6265 items [61.1%; 95% CI, 60.6%-62.1%] vs 9279 of 13 771 items [67.4%; 95% CI 66.6%-68.2%]; *P* < .001; Cramer *V* = 0.06) (eFigure 3E in Supplement 1). A similar pattern appeared for studies with a data-monitoring committee (13 807 of 21 727 items [63.5%; 95% CI, 62.9%-64.2%] vs 13 546 of 20 392 items [66.4%; 95% CI,65.8%–67.1%]; *P* < .001; *V* = 0.03) (eFigure 3F in Supplement 1), and for trials reporting serious adverse events (63.6% [95% CI, 63.8%-64.3%] vs 65.5% [95% CI, 65.0%-66.0%]; *V* = 0.02) or deaths (63.3% [95% CI, 62.2%-64.5%] vs 65.1% [95% CI, 64.7%-65.6%]; *V* = 0.01) (eFigure 3G-H in Supplement 1). All effect sizes were negligible (*V* <0.10), suggesting limited practical importance despite statistical significance.

## Discussion

Our study found that LLMs can achieve state-of-the-art performance in evaluating CONSORT compliance through zero-shot prompting, without requiring extensive fine-tuning. LLM 3 achieved excellent performance (macro F1 score, 0.85; precision, 0.96) with high agreement with human experts (91.7%), validating the potential of LLMs for automated assessment of clinical trial reporting quality at scale.

Analyzing more than 21 000 RCTs spanning nearly 6 decades revealed several important patterns in CONSORT compliance. Much of the gain coincides with successive CONSORT updates (1996, 2001, 2010); therefore, the lower compliance we observed before 1990 almost certainly reflects the absence rather than the neglect of formal guidance in that era. While reporting quality has improved substantially since 2010, coinciding with updated CONSORT guidelines and increasing journal adoption, significant gaps persist in critical methodological details. Most concerning was the systematic underreporting of elements crucial for trial reproducibility and methodological integrity, including randomization procedures (7.5%), allocation concealment mechanisms (16.1%), and protocol access information (2.2%). These gaps limit the ability of readers to fully evaluate the validity of reported findings.

The substantial variation in compliance across medical disciplines (ranging from 35% to 63%) likely reflects differences in research culture, methodological traditions, and journal policies across specialties. Specialties with entrenched research networks (eg, urology and nephrology, critical care) outperformed areas such as pharmacology and radiology, indicating scope for targeted training and editorial oversight.

Later-phase trials were better reported than early-phase studies, but differences linked to regulatory or safety oversight (eg, FDA regulation, data-monitoring committees, serious adverse-event or mortality reporting) were small (Cramer *V* <0.10) and of doubtful practical relevance. This likely reflects the parallel and sometimes competing demands of regulatory and academic reporting rather than true differences in methodological rigor. European trials may demonstrate higher compliance due to stringent European Union clinical trial regulations, stronger enforcement of reporting guidelines by European medical journals, and well-established multinational research networks that promote standardized reporting practices.

Our findings have important implications for multiple stakeholders in the clinical research ecosystem. For journal editors and publishers, they highlight the need for more rigorous enforcement of CONSORT guidelines during peer review and editing processes, particularly for consistently underreported items.

For researchers, our results identify specific reporting elements that require greater attention. Educational interventions targeting these commonly missed items could improve compliance, particularly in disciplines with lower overall adherence.

For funding agencies and regulatory bodies, our findings suggest that regulatory oversight alone does not ensure comprehensive reporting. Integration of academic reporting standards into regulatory requirements could help bridge this gap.

### Limitations

Although our study has numerous strengths, several limitations warrant consideration. First, while we mitigated LLM hallucination risks through confidence-based filtering and expert validation, further refinement of uncertainty quantification methods is needed. Second, our analysis was limited to open-access articles, which may not be representative of all published RCTs. Third, our assessment focused on the presence of reporting elements rather than their quality or accuracy. Fourth, evolving reporting standards over time may affect the interpretation of temporal trends, although our methods attempted to account for this by evaluating articles against consistent criteria. Finally, our automated assessment interrogated only the principal results manuscript for each RCT. In many contemporary trials, detailed methods are reported in separate protocol or rationale articles or made available exclusively on trial-registry websites. Because items 3b, 6b, 7b, and 14b were dropped to avoid systematic false negatives, overall model performance is probably somewhat inflated; future work will use a 2-stage prompt (first detect whether the event occurred, then evaluate reporting) so these critical items can be retained.

## Conclusions

In this systemic review study of 21 041 RCT reports, a zero-shot LLM accurately quantified CONSORT adherence and uncovered steady, yet incomplete, improvements in reporting since the guideline’s introduction. These findings suggest that automated LLM auditing offers a scalable path to monitor transparency across the biomedical literature and, if embedded in journal or registry workflows, could give authors, editors, and funders real-time feedback that accelerates progress toward fully reproducible clinical research.

## Notes

Supplement 1. **eTable 1.** CONSORT Item Definitions, Corresponding Item Numbers, and the Sections to Which They Typically Belong **eMethods.** Comprehensive Pipeline for RCT Data Acquisition and Processing **eFigure 1.** Zero-Shot Prompting Framework for CONSORT Compliance Assessment **eTable 2. **Mapping of Biomedical Specialties to the 4 Higher-Level Categories Used in All Discipline-Level Analyses **eTable 3. **Detailed Model Performance Metrics for Individual CONSORT Items **eFigure 2. **Validation of LLM Assessment Against Human Expert Evaluation **eTable 4.** Run-to-Run Stability of LLM 3 Zero-Shot Model on the CONSORT-TM Benchmark **eTable 5.** Model Performance Metrics Stratified by Confidence Levels **eFigure 3.** CONSORT Reporting Compliance Stratified by Trial Characteristics

Supplement 2.
Data Sharing Statement
