# Supplementary material for: Large Language Model Analysis of Reporting Quality of Randomized Clinical Trial Articles: A Systematic Review
Source: JAMA Netw Open. 2025 Aug 28;8(8):e2529418. doi: 10.1001/jamanetworkopen.2025.29418 (PMC12395317; doi:10.1001/jamanetworkopen.2025.29418)
Supplement: Supplement 1. — eTable 1. CONSORT Item Definitions, Corresponding Item Numbers, and the Sections to Which They Typically Belong eMethods. Comprehensive Pipeline for RCT Data Acquisition and Processing eFigure 1. Zero-Shot Prompting Framework for CONSORT Compliance Assessment eTable 2. Mapping of Biomedical Specialties to the 4 Higher-Level Categories Used in All Discipline-Level Analyses eTable 3. Detailed Model Performance Metrics for Individual CONSORT Items eFigure 2. Validation of LLM Assessment Against Human Expert Evaluation eTable 4. Run-to-Run Stability of LLM 3 Zero-Shot Model on the CONSORT-TM Benchmark eTable 5. Model Performance Metrics Stratified by Confidence Levels eFigure 3. CONSORT Reporting Compliance Stratified by Trial Characteristics [file jamanetwopen-e2529418-s001.pdf]

# Supplemental Online Content

Srinivasan A, Berkowitz J, Friedrich N, Kivelson S, Tatonetti NP. The reporting quality of randomized clinical trial articles: a systematic review. *JAMA Netw Open.* 2025;8(8):e2529418. doi:10.1001/jamanetworkopen.2025.29418

**eTable 1.** CONSORT Item Definitions, Corresponding Item Numbers, and the Sections to Which They Typically Belong

**eMethods.** Comprehensive Pipeline for RCT Data Acquisition and Processing

**eFigure 1.** Zero-Shot Prompting Framework for CONSORT Compliance Assessment

**eTable 2.** Mapping of Biomedical Specialties to the 4 Higher-Level Categories Used in All Discipline-Level Analyses

**eTable 3.** Detailed Model Performance Metrics for Individual CONSORT Items

**eFigure 2.** Validation of LLM Assessment Against Human Expert Evaluation

**eTable 4.** Run-to-Run Stability of LLM 3 Zero-Shot Model on the CONSORT-TM Benchmark

**eTable 5.** Model Performance Metrics Stratified by Confidence Levels

**eFigure 3.** CONSORT Reporting Compliance Stratified by Trial Characteristics

This supplemental material has been provided by the authors to give readers additional information about their work.

© 2025 Srinivasan A et al. *JAMA Network Open*.

## eTable 1. CONSORT Item Definitions, Corresponding Item Numbers, and the Sections to Which They Typically Belong

| Criteria | Criteria Section | Criteria Definition |
|---|---|---|
| **1a** | Title and abstract | Identification as a randomized trial in the title |
| **2a** | Introduction | Scientific background and explanation of rationale |
| **2b** | Introduction | Specific objectives or hypotheses |
| **3a** | Methods | Description of trial design (such as parallel, factorial) including allocation ratio |
| **3b** | Methods | Important changes to methods after trial commencement (such as eligibility criteria), with reasons |
| **4a** | Methods | Eligibility criteria for participants |
| **4b** | Methods | Settings and locations where the data were collected |
| **5** | Methods | The interventions for each group with sufficient details to allow replication, including how and when they were actually administered |
| **6a** | Methods | Completely defined pre-specified primary and secondary outcome measures, including how and when they were assessed |
| **6b** | Methods | Any changes to trial outcomes after the trial commenced, with reasons |
| **7a** | Methods | How sample size was determined |
| **7b** | Methods | When applicable, explanation of any interim analyses and stopping guidelines |
| **8a** | Methods | Method used to generate the random allocation sequence |
| **8b** | Methods | Type of randomisation; details of any restriction (such as blocking and block size) |
| **Criteria** | **Criteria Section** | **Criteria Definition** |
| **9** | Methods | Mechanism used to implement the random allocation sequence (such as sequentially numbered containers), describing any steps taken to conceal the sequence until interventions were assigned |
| **10** | Methods | Who generated the random allocation sequence, who enrolled participants, and who assigned participants to interventions |
| **11a** | Methods | If done, who was blinded after assignment to interventions (for example, participants, care providers, those assessing outcomes) and how |
| **12a** | Methods | Statistical methods used to compare groups for primary and secondary outcomes |
| **12b** | Methods | Methods for additional analyses, such as subgroup analyses and adjusted analyses |

© 2025 Srinivasan A et al. *JAMA Network Open*.

| | | |
|---|---|---|
| **13a** | Results | For each group, the numbers of participants who were randomly assigned, received intended treatment, and were analysed for the primary outcome |
| **13b** | Results | For each group, losses and exclusions after randomisation, together with reasons |
| **14a** | Results | Dates defining the periods of recruitment and follow-up |
| **14b** | Results | Why the trial ended or was stopped |
| **15** | Results | A table showing baseline demographic and clinical characteristics for each group |
| **17a** | Results | For each primary and secondary outcome, results for each group, and the estimated effect size and its precision (such as 95% confidence interval) |
| **18** | Results | Results of any other analyses performed, including subgroup analyses and adjusted analyses, distinguishing pre-specified from exploratory |
| **19** | Results | All important harms or unintended effects in each group |
| **20** | Results | Trial limitations, addressing sources of potential bias, imprecision, and, if relevant, multiplicity of analyses |
| **21** | Discussion | Generalisability (external validity, applicability) of the trial findings |
| **22** | Discussion | Interpretation consistent with results, balancing benefits and harms, and considering other relevant evidence |
| **23** | Discussion | Registration number and name of trial registry |
| **24** | Other information | Where the full trial protocol can be accessed, if available |
| **25** | Other information | Sources of funding and other support (such as supply of drugs), role of funders |

© 2025 Srinivasan A et al. *JAMA Network Open*.

## eMethods. Comprehensive Pipeline for RCT Data Acquisition and Processing

Our systematic approach to identifying, collecting, and processing RCT publications involved a multi-step pipeline designed to ensure comprehensive coverage and high-quality data extraction:

*Step 1: Systematic RCT Identification*
We queried the PubMed database via the Entrez API using Python with the following search criteria: ("randomized controlled trial"[Publication Type]) AND ("humans"[MeSH Terms]) AND ("1966/01/01"[Date - Publication] : "2024/12/31"[Date - Publication]). This query identified human randomized controlled trials published between 1966 and 2024, providing a comprehensive initial dataset for our analysis.

*Step 2: URL Extraction*
For all identified RCTs, we extracted their corresponding URLs from the PubMed database using the Entrez API. These URLs served as the source for accessing the full-text articles in subsequent steps.

*Step 3: Full-Text Article Acquisition*
To ensure legal and open access to research articles, we restricted our downloading to NCBI-hosted open access PDFs. From this collection, we strategically sampled articles from different time periods to create a representative dataset spanning the entire study timeframe (1966-2024). This approach enabled temporal trend analysis while maintaining manageable computational requirements.

*Step 4: Metadata Enrichment*
For each downloaded full-text article, we extracted comprehensive metadata using the Semantic Scholar API. This included publication title, year, journal information, citation metrics (both total and influential citations), author information, and other bibliometric data essential for our analysis of reporting quality trends and correlations with publication characteristics.

*Step 5: Clinical Trial Registry Data Integration (for a subset of articles)*
For articles published after the establishment of ClinicalTrials.gov, we extracted NCT numbers from the article text using GPT-4 and then matched these identifiers with clinical trials registry data for publications that contained them. This enriched dataset included information on trial phase, funding source, FDA regulation status, presence of data monitoring committees, and safety outcome reporting.

This systematic data collection and processing pipeline ensured a comprehensive, representative, and metadata-rich dataset of 21,041 RCTs for our analysis, enabling robust examination of CONSORT compliance trends across time periods, disciplines, and trial characteristics.

© 2025 Srinivasan A et al. *JAMA Network Open*.

## eFigure 1. Zero-Shot Prompting Framework for CONSORT Compliance Assessment

````
SYSTEM_PROMPT = "You are a highly skilled medical research assistant with extensive knowledge of randomized
controlled trials and CONSORT guidelines. Your task is to assess whether specific randomized controlled trial
articles meet the given CONSORT criteria. For each criterion, you will analyze the article text and provide a
detailed justification for your assessment."

prompt = f"""
# Task
Your job is to assess whether the given article meets the specified CONSORT criterion and provide
justification for your assessment.
# Article

Below is the article text:

```
{json.dumps(article, indent=2)}
```

# CONSORT Criterion

The criterion being assessed is: "{criterion}: {definition}"

# Assessment

Given the criterion above, use the article text to determine whether the article meets this criterion. Think step
by step, and justify your answer.

Format your response as a JSON object with the following keys:
* criterion: str - The name of the criterion being assessed
{'* rationale: str - Your reasoning as to why the article does or does not meet that criterion' if not
is_exclude_rationale else ''}
* is_met: bool - "true" if the article meets that criterion, "false" otherwise.
* confidence: str - Either "low", "medium", or "high" to reflect your confidence in your response
"""
````

© 2025 Srinivasan A et al. *JAMA Network Open*.

## eTable 2. Mapping of Biomedical Specialties to the 4 Higher-Level Categories Used in All Discipline-Level Analyses

| Broader medical category | Biomedical Specialities |
|---|---|
| **Organ-System Clinical Care** | Cardiology; Endocrinology/Metabolism; Nephrology; Urology; Neurology; Psychiatry; Critical Care; Gastroenterology/Hepatology; Surgery; Oncology; Immunology; Infectious Disease; Orthopedics; Dermatology; Rehabilitation; Pediatrics; Geriatrics; Primary Care |
| **Diagnostic & Procedural Support** | Radiology; Anesthesiology; Pathology; Pharmacology; Toxicology |
| **Basic & Translational Science** | Biochemistry; Cell Biology; Genetics; Developmental Biology; Physiology; Pharmaceutical Science |
| **Population & Allied Health** | Public Health; Occupational Health; Nursing; Health Informatics |

© 2025 Srinivasan A et al. *JAMA Network Open*.

## eTable 3. Detailed Model Performance Metrics for Individual CONSORT Items

| Criteria | Precision | Recall | F1 Score | Accuracy |
|---|---|---|---|---|
| **1a** | 1.0 | 1.0 | 1.0 | 1.0 |
| **2a** | 1.0 | 1.0 | 1.0 | 1.0 |
| **2b** | 1.0 | 1.0 | 1.0 | 1.0 |
| **3a** | 0.98 | 0.82 | 0.89 | 0.8 |
| **3b** | 0.67 | 0.5 | 0.57 | 0.94 |
| **4a** | 1.0 | 0.98 | 0.99 | 0.98 |
| **4b** | 0.95 | 0.95 | 0.95 | 0.92 |
| **5** | 1.0 | 1.0 | 1.0 | 1.0 |
| **6a** | 1.0 | 0.9 | 0.95 | 0.9 |
| **6b** | 0.57 | 0.8 | 0.67 | 0.92 |
| **7a** | 1.0 | 0.98 | 0.99 | 0.98 |
| **7b** | 0.88 | 0.64 | 0.74 | 0.9 |
| **8a** | 0.91 | 0.79 | 0.85 | 0.78 |
| **8b** | 1.0 | 0.51 | 0.68 | 0.62 |
| **9** | 0.69 | 0.95 | 0.8 | 0.82 |
| **10** | 0.89 | 0.57 | 0.69 | 0.7 |
| **11a** | 1.0 | 0.6 | 0.75 | 0.68 |
| **12a** | 1.0 | 0.98 | 0.99 | 0.98 |
| **12b** | 0.74 | 0.69 | 0.71 | 0.68 |
| **13a** | 0.97 | 0.67 | 0.79 | 0.68 |
| **13b** | 1.0 | 0.44 | 0.61 | 0.52 |
| **14a** | 1.0 | 0.48 | 0.65 | 0.56 |
| **14b** | 0.86 | 1.0 | 0.92 | 0.98 |
| **15** | 1.0 | 0.68 | 0.81 | 0.68 |
| **17a** | 1.0 | 0.56 | 0.72 | 0.56 |
| **18** | 1.0 | 0.54 | 0.7 | 0.66 |

© 2025 Srinivasan A et al. *JAMA Network Open*.

| Criteria | Precision | Recall | F1 Score | Accuracy |
|---|---|---|---|---|
| **19** | 1.0 | 0.71 | 0.83 | 0.74 |
| **20** | 0.95 | 0.41 | 0.57 | 0.46 |
| **21** | 0.85 | 0.38 | 0.52 | 0.6 |
| **22** | 1.0 | 0.96 | 0.98 | 0.96 |
| **23** | 1.0 | 0.98 | 0.99 | 0.98 |
| **24** | 1.0 | 0.14 | 0.25 | 0.88 |
| **25** | 1.0 | 0.9 | 0.95 | 0.9 |

© 2025 Srinivasan A et al. *JAMA Network Open*.

## eFigure 2. Validation of LLM Assessment Against Human Expert Evaluation

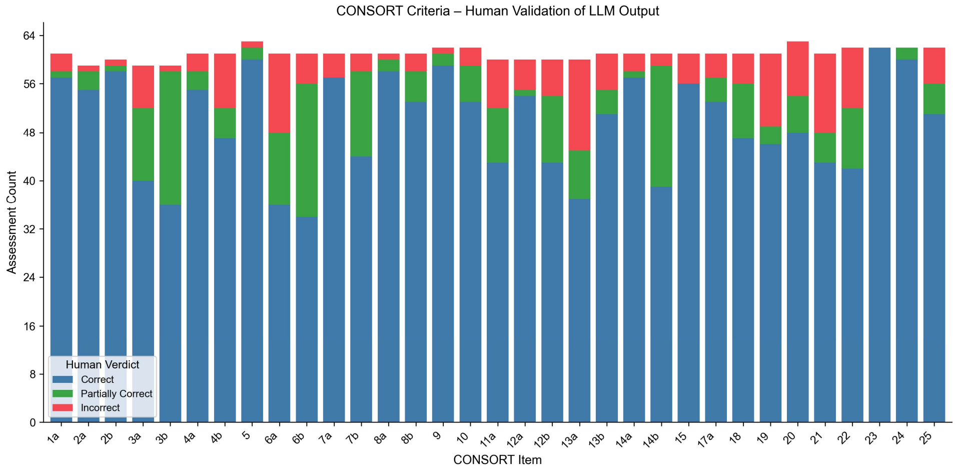


© 2025 Srinivasan A et al. *JAMA Network Open*.

## eTable 4. Run-to-Run Stability of LLM 3 Zero-Shot Model on the CONSORT-TM Benchmark

| Model | Repetition | Accuracy | Precision | Recall | F1 Score | Micro F1 Score |
|---|---|---|---|---|---|---|
| GPT-4o-mini | 1 | 0.81[0.79-0.83] | 0.97[0.95-0.98] | 0.77[0.75-0.80] | 0.86[0.84-0.87] | 0.81[0.79-0.83] |
| GPT-4o-mini | 2 | 0.81[0.79–0.83] | 0.96[0.95-0.97] | 0.79[0.76-0.80] | 0.86[0.85-0.88] | 0.82[0.79-0.83] |
| GPT-4o-mini | 3 | 0.81[0.79-0.83] | 0.96[0.94-0.97] | 0.79[0.76-0.81] | 0.86[0.85-0.88] | 0.81[0.79-0.83] |

© 2025 Srinivasan A et al. *JAMA Network Open*.

**eTable 5. Model Performance Metrics Stratified by Confidence Levels**

| Confidence | Accuracy | Precision | Recall | F1 Score | Micro F1 Score |
|---|---|---|---|---|---|
| High | 0.92 | 0.97 | 0.92 | 0.95 | 0.92 |
| Medium | 0.41 | 0.85 | 0.19 | 0.31 | 0.42 |

© 2025 Srinivasan A et al. *JAMA Network Open*.

## eFigure 3. CONSORT Reporting Compliance Stratified by Trial Characteristics

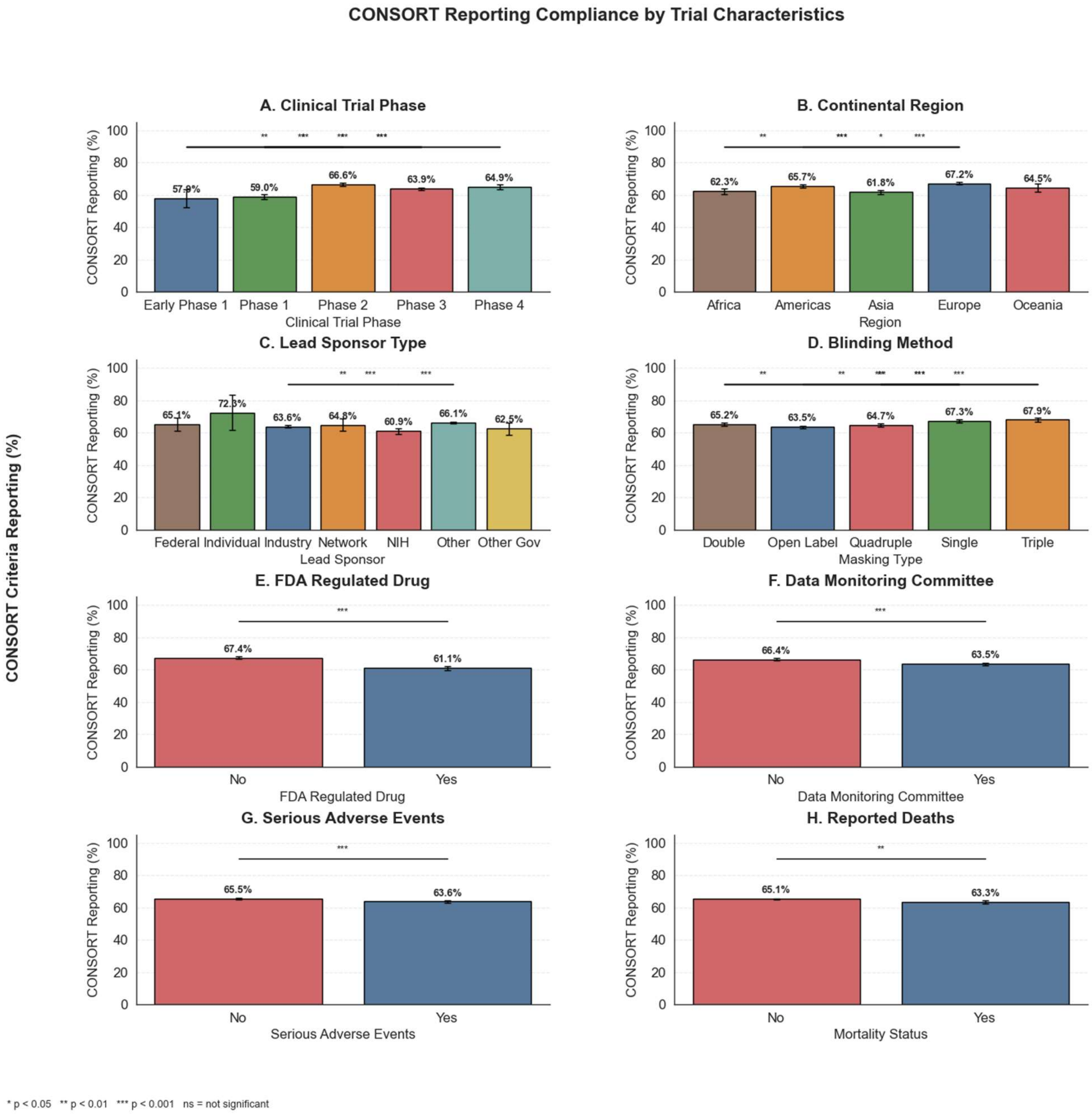


© 2025 Srinivasan A et al. *JAMA Network Open*.
