# Supplementary material for: Large Language Model Analysis of Reporting Quality of Randomized Clinical Trial Articles: A Systematic Review
Source: JAMA Netw Open. 2025 Aug 28;8(8):e2529418. doi: 10.1001/jamanetworkopen.2025.29418 (PMC12395317; doi:10.1001/jamanetworkopen.2025.29418)
Supplement: Supplement 2. — Data Sharing Statement [file jamanetwopen-e2529418-s002.pdf]

Data Sharing Statement

Srinivasan. The Reporting Quality of Randomized Clinical Trial Articles. *JAMA Netw Open*. Published August 28, 2025. doi:10.1001/jamanetworkopen.2025.29418

Data
Data available: Yes
Data types: Data (not involving human participants)
How to access data: https://huggingface.co/datasets/apoorvasrinivasan/CONSORT-21K
When available: With publication

Supporting Documents
Document types: Statistical/analytic code
How to access documents: https://github.com/tatonetti-lab/rct_consort
When available: With publication

Additional Information
Who can access the data: anyone requesting the data
Types of analyses: for any purpose
Mechanisms of data availability: freely available with/without investigator support
